# Implementation and evaluation of the 3 Wishes Project in safety-net hospitals: Protocol for a hybrid effectiveness-implementation study

**DOI:** 10.1371/journal.pone.0320843

**Published:** 2025-05-02

**Authors:** Thanh H. Neville, Anne Walling, Neil S. Wenger, Brian S. Mittman, Chi-Hong Tseng, Dong Chang, Deborah Cook, Carolyn Jennifer Marentes Ruiz, Hope Cassano, Emily Beers, Rucha Gadgil, Nader Kamangar, Nancy Blake, Derjung M. Tarn

**Affiliations:** 1 David Geffen School of Medicine at UCLA, Los Angeles, California, United States of America; 2 VA Greater Los Angeles Healthcare System, Los Angeles, California, United States of America; 3 Department of Research and Evaluation, Kaiser Permanente, Oakland, California, United States of America; 4 Los Angeles General Medical Center, Los Angeles, California, United States of America; 5 Departments of Medicine, Clinical Epidemiology and Biostatistics, McMaster University, Hamilton, Ontario, Canada; 6 Harbor-UCLA Medical Center, Torrance, California, United States of America; 7 Olive View-UCLA Medical Center, Los Angeles, California, United States of America; The Hong Kong Polytechnic University, HONG KONG

## Abstract

**Background:**

Palliative care interventions in the intensive care unit have been shown to improve communication and the quality of death and dying. However, few interventions have been implemented in safety-net hospitals (SNHs), which provide healthcare to low income and uninsured patients. The 3 Wishes Project (3WP) is a low-cost intervention that aims to enhance compassionate end-of-life (EOL) experiences by empowering the clinical team to elicit and fulfill small wishes for patients who are dying in the ICU.

**Objective:**

To implement and evaluate the 3WP in three SNHs in Los Angeles.

**Methods:**

We conduct a pragmatic cluster-randomized stepped wedge type 2 hybrid effectiveness-implementation study to implement and evaluate the effect of the 3WP (compared to usual care) on quality of EOL ICU care, bereaved families’ psychological symptoms, and clinician burnout. Prior to implementation, interviews with stakeholders from each hospital will refine the implementation strategy. Starting 6–10 months prior to 3WP implementation at each site and continuing throughout the study, we will survey bereaved families once 3 months after each patient’s death. Surveys will query: EOL care, anxiety, depression, and post-traumatic stress disorder (PTSD). Each SNH ICU starts the 3WP at a randomly assigned time that is staggered by 2 months. Nurses are surveyed on burnout before implementation, 6 months, and 12 months after 3WP implementation. Semi-structured interviews are conducted with 10–12 family members per SNH who received 3WP and 10–12 nurses per SNH a year after implementation. We will use the RE-AIM (Reach, Effectiveness, Adoption, Implementation, and Maintenance) framework to guide a mixed-methods evaluation of the 3WP implementation in SNHs.

**Conclusion:**

We hypothesize that it will be feasible to adapt and implement the 3WP in SNHs. We will evaluate how the 3WP improves EOL care in this setting and provide valuable insight regarding the adaptations necessary to tailor palliative care interventions for SNH ICUs.

**Trial registration:**

ClinicalTrials.gov NCT06277310.

## 1. Introduction

Palliative care interventions in the ICU have been shown to increase family satisfaction in the quality of death and dying [1], improve communication [2–4]. and decrease bereaved families’ psychological symptoms [5]. Compassionate end-of-life (EOL) care is foundational to medicine, but few palliative care interventions have been tested or implemented in safety-net hospitals (SNHs). SNHs are public hospitals that provide disproportionate amounts of healthcare to low-income and uninsured patients [6,7] and have less discretionary funds for innovative initiatives. Patients often present to SNHs with such advanced disease that it is often difficult to establish clinician-patient rapport prior to EOL conversations. A legacy of medical neglect and limited access to care can create distrust [8,9]. Language barriers, low health literacy, and cultural differences can also make it difficult for clinicians to adequately convey empathy and support [9–12]. Families of patients who die in intensive care units (ICUs) often suffer from anxiety and depression [13–16], post-traumatic stress disorder (PTSD) [14–16], and complicated grief [17], all of which can be exacerbated by social inequality [18–20]. Although it is recognized that EOL care in ICUs needs to be improved [21,22], no studies have evaluated palliative care interventions in the ICU in SNHs.

The 3 Wishes Project (3WP) improves the EOL experience by empowering healthcare workers (HCWs) and family members to elicit and fulfill modest wishes for critically ill patients who are dying in the hospital [23–25]. These small “wishes” are simply acts of kindness (often non-clinical) that are implemented to improve the patient’s and families’ EOL experience. Examples have included: playing the patient’s favorite music, providing the patient with a non-hospital blanket, allowing the patient to spend their final moment outdoors, giving the patient a taste of their favorite food or drink, decorating the room with the patient’s favorite memorabilia, and providing the bereaved family with keepsakes. The 3WP has been shown to enrich interpersonal connections among patients, family members and clinicians, ease family grief, and enhance clinician satisfaction [23–31]. The direct cost of wishes averages $30 per patient, and the 3WP has been shown to be acceptable and valuable to families, clinicians, and hospital leadership in a small sample of high-resourced hospitals [24,28,32]. However, data are lacking about strategies for successfully implementing 3WP in low-resourced hospitals, where patient’s experience of EOL and palliative care may be influenced by low health literacy, heterogeneous cultural perspectives, and limited English proficiency [33,34].

Because the 3WP has been shown to enhance clinician satisfaction, there is also potential for it to reduce burnout in HCWs, particularly nurses. The U.S. healthcare system is experiencing a critical shortage of nurses, and one of the most common reasons for the exodus of critical care nurses from their profession is burnout [35–37]. In our prior studies, 3WP was most frequently initiated and implemented by nurses (up to 75.7%) [24,38]. EOL care is one of the most emotionally and psychologically challenging aspects of bedside care [39–42]. As such, by providing structure and resources, and empowering SNH nurses to elicit and implement final wishes for dying patients and their families, the 3WP can potentially enhance the meaningfulness of work, decrease emotional isolation and depersonalization, and destigmatize the natural emotional responses to EOL care in the ICU [27].

We hypothesize that with the help of a tailored implementation strategy, which includes training materials and tools for bedside implementation and unit-level engagement, the 3WP can be implemented within the constraints of a resource-poor setting, improve nursing burnout, and improve the quality of EOL ICU care for dying SNH patients. In this paper, we describe a pragmatic type 2 hybrid effectiveness-implementation study (NIH R01NR020773), in which the 3WP is implemented using a cluster-randomized stepped-wedge study design at 3 SNHs. Using mixed methods, we will measure the effects of 3WP on two key clinical outcomes: a) the quality of EOL ICU care and bereaved families’ psychological symptoms, and b) nurse burnout as compared to usual care. We will also measure key implementation outcomes guided by the RE-AIM framework (Reach, Effectiveness, Adoption, Implementation, and Maintenance) [43] via quantitative measurement of reach and adoption as well as qualitative interviews with families and clinicians to measure additional implementation processes and outcomes. We hypothesize that the 3WP can be implemented in resource-poor settings and will improve the quality of EOL ICU care for dying patients and for nurses in SNHs.

## 2. Clinical intervention: The 3 Wishes Project (3WP)

The 3WP is a palliative care intervention in which clinicians, most commonly the patient’s bedside nurse, elicit and fulfill small wishes for critically ill patients who are dying in the ICU [23]. Our previous multi-center study in demonstrated the value, affordability, and sustainability of the 3WP at four tertiary academic centers in North America [24]. Examples of wishes include playing the patient’s favorite music, creating keepsakes for the family, and personalizing the environment with decorations and items from home [29]. The number and type of wishes depends on the needs of the patient/family, and an average of 3.4 wishes (range 1–10) were implemented per patient at an average cost of approximately $30 per patient.

## 3. Methods

### 3.1. Overview

**Fig 1:**
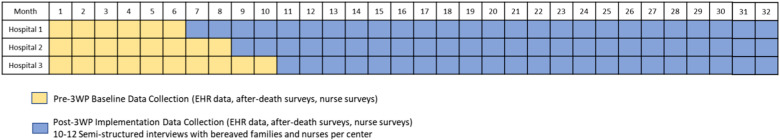
Stepped-wedge study design.

Prior to implementing the 3WP intervention, we will refine the components of the implementation strategy (below) by engaging stakeholders at each SNH. We will then use a cluster-randomized stepped-wedge type 2 hybrid effectiveness-implementation design to implement the 3WP and assess its effectiveness in improving the EOL experience for SNH patients and their families. One week prior to intervention roll-out, we will conduct training sessions with ICU nurses that go over 3WP objectives, ways to introduce the 3WP to patients and families, and resources available. We will establish a tailored inventory of items that can be used in wish fulfillment based on prior experience and suggestions from each SNH. A research assistant at each SNH will assist with logistics, support nurses with wish implementation if needed, and collect data. Each SNH ICU (cluster) begins in the control phase and will start 3WP implementation at a randomly assigned time (wedge) (Fig 1). Implementation at each center will be staggered by 2 months. This design is useful in situations where 1) there is evidence of efficacy [23,24,26–30] and there is more potential for the intervention to do good than harm, 2) the traditional experimental randomization may be viewed as inappropriate given the nature of the intervention (a stepped-wedge design would allow all safety-net ICUs to initiate the 3WP), and 3) there are logistical constraints to randomization (3WP is a unit-level intervention and randomization at the patient level will likely result in contamination) [44,45].

Each safety-net hospital will begin in the control phase and will implement the 3 Wishes Program at a randomly assigned time.

After 2.5 years, we will conclude survey collection and remove the research assistant who was available to assist with wish implementation. We will continue to provide monetary resources to purchase wish items and ask clinicians to continue documenting on 3WP during a 12-month maintenance period. Data on expenses and resource utilization will be collected during this period to provide hospital leadership with an estimation of 3WP cost when the detailed evaluation is complete and there is no on-site research assistant to facilitate this palliative care intervention. (IRB #23-0617).

### 3.2. Setting

The 3 SNHs in this study are part of the Los Angeles County Department of Health Services (DHS), which is the second largest municipal health system in the nation and provides care to patients regardless of their ability to pay. Over 50% of patients do not speak English and about 10% are unhoused. The patient population is 55–80% LatinX, 20% Asian, and 10% black, with many living individuals below the federal poverty level. Although all 3 hospitals serve poor, under- or uninsured, and non-English-speaking patients, they are of different sizes, have different geographical catchment areas, and patient populations. Because the mortality is the highest in medical ICU’s, this is where we will implement and evaluate the 3WP in these 3 Los Angeles DHS SNHs. Of note, hospital 1 only has one combined medical-surgical ICU.

### 3.3. Participants

To participate in the 3WP, a patient must be: 1) a critically ill adult in the ICU and 2) have a high risk of dying during the hospitalization as judged by the ICU physician, or a decision has been made to withdraw or withhold life support in anticipation of death. Family member(s) and/or the person listed as the patient’s designated decision maker (hereafter referred as family) can be included in wish solicitation and fulfillment. Based on our prior experience, we anticipate less than 10% of patients will be able to express their wishes due to critical illness [28,46]. Eligibility identification and wish solicitation and fulfillment are performed by any clinician, typically the ICU nurse and confirmed with the ICU physician(s). The 3WP is not meant to substitute a goals of care conversation, and as such, the nurse who wants to implement 3WP would confirm with the ICU physician that the patient is an appropriate candidate, and that the family is aware of their poor prognosis.

Regardless of whether the patient participates in the 3WP, families of all patients who die in the ICU will be contacted to complete an after-death survey. “Families” are those who are listed as the emergency contact or next of kin in the patient’s medical record, must be ≥ 18years old, and English- or Spanish-speaking. We anticipate that contact information will be missing for 5–10% of patients (i.e., due to homelessness). Additionally, families (10 per site) of patients who participated in the 3WP will also be invited for semi-structured interviews. All nurses (approximately 250 nurses across 3 ICUs) who work in the ICU in which the 3WP is being implemented will be eligible to complete surveys regarding burnout and compassion before and after 3WP implementation. Ten nurses at each site who have implemented the 3WP for their patients will also be invited to semi-structured interviews.

### 3.4. Implementation strategy refinement with stakeholder engagement

The 3WP implementation strategy is operationalized as having a series of core functions and customizable forms based on a conceptual model by Jolles et al. [47] (Table 1). In this framework, the core functions are the primary purposes that the implementation approach seeks to achieve, while the forms are the specific activities that can be customized with input from each SNH to operationalize the core functions. The core functions are: 1) methods for engagement and training; 2) tools for bedside 3WP implementation; 3) a method for documentation and tracking, and 4) tools to encourage unit-level engagement. Strategies to implement 3WP will be individualized for each SNH.

**Table 1:** The 3WP implementation function and form matrix.

	Core functions	Forms (examples only, to be tailored by SNH)
Tools for initial engagement and training	Familiarize clinicians with 3WP mission and goals Train clinicians with 3WP initiation and bedside implementation process Establish on-the-ground champions	PowerPoint presentation to introduce 3WP to ICU clinicians Teaching videos on how to offer 3WP to patients/families Recommendations to establish day and night shift nurse champions
Tools for bedside wish elicitation and fulfillment	Decrease burden of bedside implementation Ensure equitable 3WP offering Provide information to patients/families on the program Guide elicitation and implementation of wishes that personalize the patient’s death and create positive memories for family	Script for nurses on how to introduce the 3WP Badge buddies to delineate steps Announce eligible patients in daily multidisciplinary rounds or morning huddles List of inventory items (blankets, keepsake supplies, gift certificates to local stores, etc.) & recommendations to stock in accessible area 3WP informational brochures for patients/families Examples of possible wishes to suggest/implement
Tools to document and track	Communicate 3WP implementation with other members of the clinical team Track 3WP implementation (patients, families, and clinicians who participated, wishes achieved and cost)	Templates to assist with EHR documentation Online database to record patients who participated, the wishes fulfilled, and cost
Tools for unit-level engagement and recognition	Provide recognition and motivation for the team’s work Communicate progress and address questions and concerns	Templates for emails and flyers/posters summarizing accomplishments Guidance for conducting quarterly meetings to share stories and address questions and/or concerns

The forms listed in Table 1 are based on our prior experience but will be shaped by semi-structured interviews with stakeholders to best conform to the local needs and context of each hospital. Site lead investigators will identify key stakeholders at each SNH and semi-structured interviews will be conducted with 15–20 (5–7 per SNH) nurses and physicians during the pre-implementation phase. Interview questions are designed to identify actionable contextual factors using the Consolidated Framework for Implementation [48]. They address characteristics of the proposed implementation approach (usefulness of components, the need for additional forms or core functions), the optimal process (establishing 3WP champions, documenting participants and wishes, sharing 3WP achievements with the clinical team), the outer setting (SNH priorities, including concerns about new interventions and resource constraints), inner setting (ICU infrastructure, compatibility with workflow, space for 3WP inventory), and the characteristics of the individuals involved (diverse patient/family needs, nursing time constraints). At least 3 members of the research team will listen to interview recordings and independently perform content analysis [49,50] to identify all forms suggested by participants. The researchers will discuss findings and produce a complete core function/form matrix listing all suggested forms by core function.

### 3.5. Data collection

#### 3.5.1. Electronic health record (EHR) abstraction.

Each decedent will be characterized using EHR data, including the patient’s age, sex, race/ethnicity, language, insurance status, co-morbidities, and ICU and hospital length of stay. We will also note palliative care consultations, utilization of life-sustaining treatments (mechanical ventilation, vasopressors, or dialysis), and the occurrence of CPR as these factors have been shown to be associated with quality of death [51–53]. We will manually abstract occurrence of family meetings, reason for ICU admission, resuscitation status at the time death, whether the family was present at the time of death, and whether the death was expected. Expected death is defined as any physician documentation during the terminal hospitalization that the patient was terminal, had a grave prognosis, was receiving hospice care, had imminently life-threatening disease in the context of a poor prognosis, or was dying; this information was previously reliably abstracted from the medical record (reabstraction κ=0.67; 91% agreement) [54]. Double abstraction will be performed for 30 patients and a kappa will be calculated to evaluate inter-rater reliability, with appropriate changes in abstraction process or abstractor training implemented to increase inter-rater reliability if needed.

#### 3.5.2. Surveys.

An after-death survey that assesses perceptions regarding EOL care (Bereaved Family Survey (BFS)) [55] and psychological symptoms (Hospital Anxiety and Depression Scale (HADS) [56] and PTSD Checklist for DSM-5 (PCL-5) [57]) will be mailed to each family of an ICU decedent throughout the study period, 3 months after the patient’s death (both pre- and post-intervention) (Fig 1). The postal mailing will include a stamped return envelope, a phone number and email address for questions, and a letter stating that completing the survey implies consent and that there will be a $40 honorarium for completion. Surveys will be tracked using a unique code. Two weeks following the initial mailing, non-responders will be called up to 3 times (at least one attempt after 5pm and maximum two voice messages) to remind families to mail it back, or to complete it by phone or online. We are anticipating a return rate of approximately 40% based on our group’s prior experience [58,59] and other studies evaluating the quality of dying [60–62].

All nurses in participating ICUs will be invited to complete on-line surveys during the month prior to 3WP training and at 6 and 12 months after 3WP implementation. The survey will include the Maslach Burnout Inventory-Human Services Survey (MBI-HSS) [63] as well as questions regarding nurse’s age, sex, race/ethnicity, marital status, years of experience, average number of hours worked per week, and whether the nurse works day or night shift. Surveys will also ask nurses to rank 4 statements (0–4 scale) to assess the extent of implementation fidelity to core functions: 1) The introductory/training sessions provided me with the necessary instructions for 3WP implementation. 2) Available tools and materials made it easy to implement 3WP at the bedside. 3) I feel comfortable documenting 3WP implementation. 4) My ICU has embraced the 3WP as a unit-level intervention. There are approximately 250 ICU nurses across the 3 SNHs and we are anticipating 150 nurse evaluations at each time point (60% response rate). The survey will take 10–15 minutes to complete, will be compensated with a $20 gift card, and will include coded identifiers to link pre- and post-implementation surveys.

#### 3.5.3. Semi-structured interviews.

At each SNH, we will recruit 10–12 family members of patients who participated in the 3WP for semi-structured interviews (prior studies have shown that 92% of themes are identified in the first 10–12 interviews) [64,65]. Families will be contacted 10–12 weeks after the patient’s death. Interviews will be conducted in Spanish or English and will be audio-recorded. A semi-structured interview guide will be used to query family members about their experience with their loved one’s EOL care, the impact of the 3WP on their experience, any barriers/facilitators to participating in 3WP, and any influence of the 3WP on their grieving process and well-being. Interviewees will be verbally consented and receive $25 for their participation.

After 1 year of 3WP implementation, site PIs will recruit ICU nurses for interviews. Participants must have implemented the 3WP for at least one dying patient. Approximately 10–12 nurses will participate in semi-structured interviews at each SNH. Interview questions will explore whether and how the 3WP affected nurses’ sense of personal accomplishment, depersonalization, and emotional exhaustion in the care of dying patients. Interviewees will be verbally consented and receive $25 for their participation.

### 3.6. Outcomes and analysis

#### 3.6.1. Patient/family outcomes.

The primary outcome is changes in the Emotional and Spiritual Support factor on the family reported BFS after 3WP implementation. Three BFS-derived measures will be analyzed: Respectful Care and Communication (5 questions, Cronbach α = 0.82), Emotional and Spiritual Support (3 questions, Cronbach α = 0.77), and the BFS-Performance Measure (BFS-PM). The Respectful Care and Communication factor score sums 5 items about staff behavior: (1) listened to concerns; (2) provided medical treatment patient wanted; (3) were kind, caring, and respectful; (4) kept family members informed about patient’s condition and treatment; and (5) attended to personal care needs. The Emotional and Spiritual Support (ESS) factor score sums 3 items about whether staff provided: (1) enough emotional support before death; (2) enough spiritual support; and (3) enough emotional support after death. Based on our prior work [31], we hypothesize that the 3WP will increase the ESS factor score. The BFS-PM, a single item asking for a global rating of EOL care, will be a secondary outcome. Secondary outcomes will also include scores on the HADS (depression and anxiety) and PCL-5 (PTSD) measures.

**Statistical analysis**. We will use an analysis approach that accounts for the stepped-wedge design of this study [66,67]. We will use mixed effects logistic and linear mixed effects models to assess the effect of the intervention on the BFS ESS factor score, BFS-PM, FS-ICU, HADS, and PCL-5. These models adjust for center effect and the confounding effects of time and 3WP implementation. With $Y_{ijt}$ as the quantitative outcome for patient i from center j at time t, the model for patient/family reported outcomes is:

$Y_{ijt}=\mu +\alpha_{j}{\;+\;\beta }_{t}+X_{ijt}\theta +\;Z_{ijt}\varphi +\in_{ijt},$

where μ is the intercept, $\gamma_{i}$ is a random center effect, $\beta_{t}$ is the fixed time effect, $X_{ijt}$ is the indicator of treatment mode for subject i cluster j at month t, θ represents the 3WP implementation effect, $Z_{ijt}$ are pre-specified patient and practice level covariates and $\in_{ijt}$ is random errors. We will also approximate the secular time trend $\beta_{t}$ linearly in time with βt. This model will accommodate unobserved center effects. All analyses will be carried out based on the intention to treat principle, which means that the treatment mode variable ($X_{ijt})$ is determined by the scheduled initiation of implementation based on randomization, but not by individual patient/family enrollment in the 3WP. Covariates, including age, sex, race/ethnicity, language, insurance status, and utilization of life-support measures, will be included in the model. We will also perform “as-treated” analyses to compare patients before implementation and patients who received 3WP after implementation and those who did not. Subgroup analyses will evaluate how outcomes are affected by race, ethnicity, and sex as a biological variable.

**Power calculations.** The primary outcome is the family-reported ESS factor score on the BFS, and the secondary endpoints include BFS-PM, FS-ICU, HADS and PCL-5. Across the 3 SNH ICUs, we estimate that 375 (90%) deaths per year will qualify for after-death surveys About 40% of families will return the survey based on our prior work [58,59]. With approximately 100 surveys returned before and 275 surveys after 3WP implementation and a 2-sided significance (α) level of 0.05, we will have 89.3% power to demonstrate the difference in the BFS ESS factor based on our preliminary data [59].

#### 3.6.2. Nurse outcomes.

Secondary outcomes include changes in nurse burnout as assessed by MBI-HSS, which consists of 22 items utilizing 7-point Likert scale responses ranging from 0 (never) to 6 (everyday) on items aimed at measuring the 3 dimensions of burnout [63]. The scores of the items in each MBI dimension will be summed; high scores for emotional exhaustion and depersonalization indicate higher levels of burnout, while high scores for personal accomplishment indicate lower levels of burnout. According to a recent meta-analysis on interventions that reduce clinician burnout [68], we anticipate that the 3WP will reduce the burnout by about 0.3–0.45 in effect size. With 150 nurse evaluations at each time point (60% response rate), we will have >85% power to detect a difference the MBI emotional exhaustion scale. This calculation conservatively assumes 0.5 correlations between before and after survey from the same nurses. We will use linear mixed effects to assess the effect of the 3WP intervention on nursing burnout, with nurses as a random effect, within the stepped-wedge study design. The analysis will control for nurse characteristics (i.e., age, sex, race, ethnicity, and years of experience) as well as the unique nurse identifier.

#### 3.6.3. Outcomes from semi-structured interviews.

We will follow PCORI methodology standards for qualitative analysis [69] to establish rigor and trustworthiness of semi-structured interviews from family members and nurses. Key methods will include performing purposive sampling of interviewees to ensure diversity, using coders from different backgrounds to increase interpretation reliability, and conducting an audit trail for the coding process. Interviews will be transcribed verbatim in the language in which they were conducted using a standardized transcription protocol [70] and imported to qualitative analytic software (*Atlas.ti* v24) for analysis. The qualitative analysis team will be an interdisciplinary team that will include bilingual Spanish speakers. The team will employ thematic analysis to analyze the data collected. The coding team will inductively develop codes describing participant perceptions and feedback regarding 3WP and will work together to develop consensus regarding a set of codes. To facilitate reliability, a codebook will be developed listing each code name, definition, and positive and negative exemplars [71]. The investigators will subsequently develop themes by examining the codes to identify patterns and relationships in the data. Themes will related to participant perceptions about the 3WP with a focus on the ability of 3WP to personalize care, the effect of 3WP on grieving, and opportunities for improvement. The coding team will share generated themes with the rest of the investigative team. Data will be examined for theoretical saturation of themes (when no new themes can be identified from the data) [72,73].

For nurse interviews, themes regarding how the 3WP affected nurses’ perceptions of how well they can provide high quality EOL care and how the 3WP affected their sense of personal accomplishment, emotional exhaustion, depersonalization, and professional morale will be identified using deductive thematic analysis [49,50]. We will also identify themes related to the feasibility and acceptability of the intervention in SNHs and suggestions for implementation improvement.

#### 3.6.4. Implementation outcomes.

The Reach, Effectiveness, Adoption, Implementation, and Maintenance (RE-AIM) framework will be used to define implementation outcomes (Table 2). *Reach:* Each month, we will obtain lists of deaths in each ICU from the bioinformatics department. This will be the denominator to determine the proportion of eligible patients who received the 3WP. Nurses will be asked to document a 3WP note in the EHR, such that we will be able to record who initiated and implemented the initiative and track the number of clinicians involved at each SNH. *Effectiveness:* The effectiveness of the 3WP in improving the EOL experience for patients/families and nurses will be evaluated by surveys and interviews (above). *Adoption.* We will compare variation in rates of 3WP patient enrollment and proportion of clinician participation across the 3 SNHs. *Implementation.* Nurses’ answers to the 4 questions on fidelity to core functions will be averaged and compared across SNHs. We will also describe categories of wishes, number of wishes, and percent of elicited wishes implemented and by whom. We will present the average cost per patient, and the number and proportion of no-cost wishes. These descriptive statistics will be compared across centers and with historical multi-center data [46]. *Maintenance.* We will trend the descriptive statistics (patients enrolled, clinicians involved, wishes implemented, cost of wishes), by month and by center. After 2.5 years, we will conclude survey collection and remove the research assistant who was available to assist with wish implementation but continue to provide monetary resources to purchase items for patients/families for an additional 12-month period. This period will represent the maintenance period and will also provide an estimation of 3WP cost when there is no on-site research assistant should the site choose to continue the intervention; these data will be provided back to 3WP leaders.

**Table 2:** Application of the RE-AIM framework to mixed method evaluation of 3WP implementation.

Domain	Existing knowledge gaps in 3WP implementation	Quantitative data	Qualitative data
Reach	Extent to which clinicians are willing to uptake 3WP as ICU initiative in SNHs Extent to which under-served patients and families are willing to accept 3W offering	% of ICU nurses involved in 3WP implementation % of eligible patients who are offered and receive 3WP Characteristics of patients who participated (demographics, insurance, clinical characteristics)	Identify factors influencing 3WP acceptability for families Identify factors influencing 3WP feasibility for nurses
Effectiveness	3WP feasibility and effectiveness has not been evaluated in SNHs Quantitative evidence of improved EOL experiences & clinician burnout Whether patient population SNHs will favorably respond to 3WP Whether 3WP has an effect on family psychological symptoms	Effect of 3WP on family’s perspective of EOL care (BFS) Effect of 3WP on family’s anxiety, depression, and PTSD Effect of 3WP on nurse burnout	Explore effect of 3WP on family’s EOL experience, satisfaction, bereavement, and psychological symptoms Explore effect of 3WP on clinician burnout, agency, stress of work
Adoption	3WP adoption as part of EOL care in SNHs unknown	% of eligible patients who receive 3WP as part of EOL care compared across the three SNHs	Ascertain how 3WP is integrated into workflow at each SNH, perceived benefits and burdens
Implementation	The extent of 3WP implementation fidelity (adherence to core functions) in SNHs is unknown; unclear if fidelity will vary across centers Unknown how categories of wishes, # of wishes/patient, cost compare to prior data	Rankings of fidelity to core functions (nurse surveys) Categories of wishes, # of wishes, when implemented, by whom, and cost/patient	Assessment of the forms (and adaptations) used to achieve core functions via nurse and leadership interviews Assessment of families’ and clinicians’ experience of 3WP
Maintenance	Extent to which 3WP becomes embedded into ongoing EOL care in SNHs is unknown	Variation in 3WP implementation over time Continuation of 3WP beyond the study period that included surveys and research assistant	Obtain clinician’s opinions on what influence 3WP maintenance Explore the factors that influence sustainability after removal of research assistant

### 3.7. Status and timeline

We have engaged stakeholders across the 3 SNH’s in individual or group interviews to refine and tailor the implementation plan (section 3.4). Thirty stakeholders participated: 4 unit directors, 11 charge nurses, 3 nurse educators, 7 physicians, 1 palliative care nurse, and 4 bedside ICU nurses). To obtain initial engagement and provide training (core function #1), stakeholders identified existing unit-specific meetings where 3WP can be promoted and formal training provided when appropriate, such as monthly staff meetings, daily shift change huddles, and multidisciplinary rounds. To facilitate bedside wish elicitation (#2), stakeholders recommended badge buddies and a script for nurses to reference, and informational brochures written in patient-preferred languages. To facilitate wish fulfillment (#2), sites recommended establishing an inventory of items to meet the cultural and spiritual needs of a majority Catholic patient population (rosaries, flameless prayer candles, Bibles). The electronic medical record was determined to be the ideal method for documenting 3WP participation (#3). Unit bulletin boards, health system-wide monthly newsletters, and computer screensavers were identified as spaces to recognize 3WP achievements and sustain unit engagement (#4).

3WP implementation occurred at the first SNH on April 1, 2024 and then staggered by 2 months for the subsequent two SNH’s. After-death surveys were first mailed to families of deceased patients on January 2, 2024 and will continue for 3.5 years (approximately ending in June 1, 2027). Survey data will be analyzed, and we anticipate that the results will be ready in 2028. The first family interview was conducted in August 2024. We anticipate that 30–36 interviews will be completed in two years and qualitative analysis of these interviews will be completed in 2027.

## 4. Discussion

This manuscript describes a protocol to implement and evaluate the 3WP, a low cost, high value palliative care intervention in 3 low-resourced hospitals. The study will be performed across 3 SNHs in the Los Angeles County healthcare system, the second largest municipal health system in the nation, and thus will provide valuable insight for implementing palliative care interventions in culturally diverse populations and safety-net settings. The intervention is low risk and there are minimal safety concerns. As a hybrid effectiveness-implementation study that utilizes mixed methods [74], our study will provide rich perspectives by integrating both quantitative and qualitative evaluations.

Our study is not without limitations. First, neither patients nor clinicians are randomized; however, this study will provide an assessment of effectiveness and implementation under real-world conditions. Participation in a randomized trial would require explicit patient and/or family member consent, which would generate confusion for dying patients and their family members about compassionate end-of-life interventions that are determined by randomization. Delayed and declined engagement would likely decrease the sample size and result in a biased patient sample. Second, because 3WP is an initiative that is dependent on nurses to voluntarily offer to their patients, it is also dependent on staffing radios and how busy the ICU is. SNHs may have more limited time and effort to devote to eliciting and implementing wishes for dying patients, although our prior experience has shown that clinicians value the ability to provide this type of enhanced care to their patients and families [28]. We will perform additionally perform an as-treated analysis, but also iteratively assess the best strategies for overcoming SNH limitations throughout the study. Third, three clusters may seem small, but this is an acceptable number of clusters for the stepped-wedge design [45,75]. Fourth, we are anticipating a survey return rate of approximately 40% from families of deceased patients. Although seemingly low, this is typical of after-death surveys and consistent with our experience and prior studies.[25,60,61,76]

In summary, we anticipate that this study will demonstrate the feasibility of implementing the 3WP in the SNH setting, and SNH patients and families will benefit from the 3WP by receiving improved quality EOL ICU care. Our proposal will provide both effectiveness evidence and implementation insights to guide future dissemination of the 3WP and other palliative care initiatives for critically ill patients in SNHs.

## Supporting information

S1 Protocol Study protocol. (PDF)

## Abbreviations

3WP: 3 Wishes Project
FG: focus group with clinicians
MBI: Maslach Burnout Inventory (nurses)
CD-RISC: Connor-Davidson Resilience Scale (nurses).

## References

[1] Curtis JR, Nielsen EL, Treece PD, Downey L, Dotolo D, Shannon SE, et al. Effect of a quality-improvement intervention on end-of-life care in the intensive care unit: a randomized trial. Am J Respir Crit Care Med. 2011;183(3):348–55. doi: 10.1164/rccm.201006-1004OC 20833820 PMC3056230

[2] Anderson WG, Puntillo K, Cimino J, Noort J, Pearson D, Boyle D, et al. Palliative Care Professional Development for Critical Care Nurses: A Multicenter Program. Am J Crit Care. 2017;26(5):361–71. doi: 10.4037/ajcc2017336 28864431

[3] Higginson IJ, Koffman J, Hopkins P, Prentice W, Burman R, Leonard S, et al. Development and evaluation of the feasibility and effects on staff, patients, and families of a new tool, the Psychosocial Assessment and Communication Evaluation (PACE), to improve communication and palliative care in intensive care and during clinical uncertainty. BMC Med. 2013;11:213. Epub 2013/10/03. doi: 10.1186/1741-7015-11-213 24083470 PMC3850793

[4] Shelton W, Moore CD, Socaris S, Gao J, Dowling J. The effect of a family support intervention on family satisfaction, length-of-stay, and cost of care in the intensive care unit. Crit Care Med. 2010;38(5):1315–20. doi: 10.1097/CCM.0b013e3181d9d9fe 20228678

[5] Lautrette A, Darmon M, Megarbane B, Joly LM, Chevret S, Adrie C, et al. A communication strategy and brochure for relatives of patients dying in the ICU. N Engl J Med. 2007;356(5):469–78. doi: 10.1056/NEJMoa063446 17267907

[6] Baxter RJ, Mechanic RE. The status of local health care safety nets. Health Aff (Millwood). 1997;16(4):7–23. doi: 10.1377/hlthaff.16.4.7 9248145

[7] Zwanziger J, Khan N. Safety-net hospitals. Med Care Res Rev. 2008;65(4):478–95. doi: 10.1177/1077558708315440 18640951

[8] Kayser K, DeMarco RF, Stokes C, DeSanto-Madeya S, Higgins PC. Delivering palliative care to patients and caregivers in inner-city communities: challenges and opportunities. Palliat Support Care. 2014;12(5):369–78. doi: 10.1017/S1478951513000230 24153017

[9] Lewis JM, DiGiacomo M, Currow DC, Davidson PM. Dying in the margins: understanding palliative care and socioeconomic deprivation in the developed world. J Pain Symptom Manage. 2011;42(1):105–18. doi: 10.1016/j.jpainsymman.2010.10.265 21402460

[10] Lyckholm LJ, Coyne PJ, Kreutzer KO, Ramakrishnan V, Smith TJ. Barriers to effective palliative care for low-income patients in late stages of cancer: report of a study and strategies for defining and conquering the barriers. Nurs Clin North Am. 2010;45(3):399–409. doi: 10.1016/j.cnur.2010.03.007 20804885

[11] Nedjat-Haiem FR, Carrion IV. Assessing Challenges in End-of-Life Conversations With Patients Utilizing a Public Safety-Net Health Care System. Am J Hosp Palliat Care. 2015;32(5):528–36. doi: 10.1177/1049909114530550 24752233

[12] Rhodes RL, Xuan L, Paulk ME, Stieglitz H, Halm EA. An examination of end-of-life care in a safety net hospital system: a decade in review. J Health Care Poor Underserved. 2013;24(4):1666–75. doi: 10.1353/hpu.2013.0179 PubMed . 24185162 PMC7292336

[13] Pochard F, Darmon M, Fassier T, Bollaert P-E, Cheval C, Coloigner M, et al. Symptoms of anxiety and depression in family members of intensive care unit patients before discharge or death. A prospective multicenter study. J Crit Care. 2005;20(1):90–6. doi: 10.1016/j.jcrc.2004.11.004 16015522

[14] Siegel MD, Hayes E, Vanderwerker LC, Loseth DB, Prigerson HG. Psychiatric illness in the next of kin of patients who die in the intensive care unit. Crit Care Med. 2008;36(6):1722–8. doi: 10.1097/CCM.0b013e318174da72 18520637

[15] Kross EK, Engelberg RA, Gries CJ, Nielsen EL, Zatzick D, Curtis JR. ICU care associated with symptoms of depression and posttraumatic stress disorder among family members of patients who die in the ICU. Chest. 2011;139(4):795–801. doi: 10.1378/chest.10-0652 20829335 PMC3071273

[16] Gries CJ, Engelberg RA, Kross EK, Zatzick D, Nielsen EL, Downey L, et al. Predictors of symptoms of posttraumatic stress and depression in family members after patient death in the ICU. Chest. 2010;137(2):280–7. doi: 10.1378/chest.09-1291 19762549 PMC2816640

[17] Kentish-Barnes N, Chaize M, Seegers V, Legriel S, Cariou A, Jaber S, et al. Complicated grief after death of a relative in the intensive care unit. Eur Respir J. 2015;45(5):1341–52. doi: 10.1183/09031936.00160014 25614168

[18] Ribeiro WS, Bauer A, Andrade MCR, York-Smith M, Pan PM, Pingani L, et al. Income inequality and mental illness-related morbidity and resilience: a systematic review and meta-analysis. Lancet Psychiatry. 2017;4(7):554–62. Epub 2017/05/30; doi: 10.1016/S2215-0366(17)30159-1 PubMed 28552501

[19] Pickett KE, Wilkinson RG. Inequality: an underacknowledged source of mental illness and distress. Br J Psychiatry. 2010;197(6):426–8. doi: 10.1192/bjp.bp.109.072066 21119145

[20] Wilkinson R, Pickett K. Inequality and mental illness. Lancet Psychiatry. 2017;4(7):512–3. doi: 10.1016/S2215-0366(17)30206-7 Epub 2017/05/30; 28552499

[21] Hanson LC, Danis M, Garrett J. What is wrong with end-of-life care? Opinions of bereaved family members. J Am Geriatr Soc. 1997;45(11):1339–44. doi: 10.1111/j.1532-5415.1997.tb02933.x 9361659

[22] Teno JM, Clarridge BR, Casey V, Welch LC, Wetle T, Shield R, et al. Family perspectives on end-of-life care at the last place of care. JAMA. 2004;291(1):88–93. doi: 10.1001/jama.291.1.88 14709580

[23] Cook D, Swinton M, Toledo F, Clarke F, Rose T, Hand-Breckenridge T, et al. Personalizing death in the intensive care unit: the 3 Wishes Project: a mixed-methods study. Ann Intern Med. 2015;163(4):271–9. doi: 10.7326/M15-0502 26167721

[24] Vanstone M, Neville TH, Clarke FJ, Swinton M, Sadik M, Takaoka A, et al. Compassionate End-of-Life Care: Mixed-Methods Multisite Evaluation of the 3 Wishes Project. Ann Intern Med. 2020;172(1):1–11. doi: 10.7326/M19-2438 31711111

[25] Neville TH, Taich Z, Walling AM, Bear D, Cook DJ, Tseng C-H, et al. The 3 Wishes Program Improves Families’ Experience of Emotional and Spiritual Support at the End of Life. J Gen Intern Med. 2023;38(1):115–21. doi: 10.1007/s11606-022-07638-7 35581456 PMC9113739

[26] Vanstone M, Toledo F, Clarke F, Boyle A, Giacomini M, Swinton M, et al. Narrative medicine and death in the ICU: word clouds as a visual legacy. BMJ Support Palliat Care. 2016:bmjspcare-2016-001179. doi: 10.1136/bmjspcare-2016-001179 27884867

[27] Vanstone M, Sadik M, Smith O, Neville TH, LeBlanc A, Boyle A, et al. Building organizational compassion among teams delivering end-of-life care in the intensive care unit: The 3 Wishes Project. Palliat Med. 2020;34(9):1263–73. doi: 10.1177/0269216320929538 32519615

[28] Neville TH, Agarwal N, Swinton M, Phung P, Xu X, Kao Y, et al. Improving End-of-Life Care in the Intensive Care Unit: Clinicians’ Experiences with the 3 Wishes Project. J Palliat Med. 2019;22(12):1561–7. doi: 10.1089/jpm.2019.0135 31274366

[29] Neville TH, Clarke F, Takaoka A, Sadik M, Vanstone M, Phung P, et al. Keepsakes at the End of Life. J Pain Symptom Manage. 2020;60(5):941–7. doi: 10.1016/j.jpainsymman.2020.06.011 32574658

[30] Swinton M, Giacomini M, Toledo F, Rose T, Hand-Breckenridge T, Boyle A, et al. Experiences and Expressions of Spirituality at the End of Life in the Intensive Care Unit. Am J Respir Crit Care Med. 2017;195(2):198–204. doi: 10.1164/rccm.201606-1102OC 27525361

[31] Neville TH, Taich Z, Walling AM, Bear D, Cook DJ, Tseng C-H, et al. The 3 Wishes Program Improves Families’ Experience of Emotional and Spiritual Support at the End of Life. J Gen Intern Med. 2022; Epub 2022/05/18. doi: 10.1007/s11606-022-07638-7 35581456 PMC9113739

[32] Swinton ME, Phung P, Neville TH, Takaoka A, Smith OM, Baker A, et al. Hospital Leadership Perspectives on the Value of the 3 Wishes Project: A Qualitative Descriptive Study. BMJ Leader. 2021;5:87–92.

[33] Bazargan M, Cobb S, Assari S, Kibe LW. Awareness of Palliative Care, Hospice Care, and Advance Directives in a Racially and Ethnically Diverse Sample of California Adults. Am J Hosp Palliat Care. 2021;38(6):601–9. doi: 10.1177/1049909121991522 33535787

[34] Cain CL, Surbone A, Elk R, Kagawa-Singer M. Culture and Palliative Care: Preferences, Communication, Meaning, and Mutual Decision Making. J Pain Symptom Manage. 2018;55(5):1408–19. doi: 10.1016/j.jpainsymman.2018.01.007 29366913

[35] Stechmiller JK. The nursing shortage in acute and critical care settings. AACN Clin Issues. 2002;13(4):577–84. doi: 10.1097/00044067-200211000-00011 12473920

[36] Steinbrook R. Nursing in the crossfire. N Engl J Med. 2002;346(22):1757–66. doi: 10.1056/NEJM200205303462225 12037165

[37] Bleich MR, Hewlett PO, Santos SR, Rice RB, Cox KS, Richmeier S. Analysis of the nursing workforce crisis: a call to action. Am J Nurs. 2003;103(4):66–74. doi: 10.1097/00000446-200304000-00025 12677128

[38] Neville TH, Bear DK, Kao Y, Xu XS, Hjelmhaug K, Quebral D, et al. End-of-Life Care During the Coronavirus Disease 2019 Pandemic: The 3 Wishes Program. Crit Care Explor. 2021;3(10):e549. doi: 10.1097/CCE.0000000000000549 34651136 PMC8505331

[39] Ong KK, Ting KC, Chow YL. The trajectory of experience of critical care nurses in providing end-of-life care: A qualitative descriptive study. J Clin Nurs. 2018;27(1–2):257–68. doi: 10.1111/jocn.13882 28514505

[40] Jang SK, Park WH, Kim HI, Chang SO. Exploring nurses’ end-of-life care for dying patients in the ICU using focus group interviews. Intensive Crit Care Nurs. 2019;52:3–8. doi: 10.1016/j.iccn.2018.09.007 Epub 2018/10/10; 30297148

[41] Kentish-Barnes N, Morin L, Cohen-Solal Z, Cariou A, Demoule A, Azoulay E. The Lived Experience of ICU Clinicians During the Coronavirus Disease 2019 Outbreak: A Qualitative Study. Crit Care Med. 2021;49(6):e585–97. doi: 10.1097/CCM.0000000000004939 33591018

[42] Efstathiou N, Walker W. Intensive care nurses’ experiences of providing end-of-life care after treatment withdrawal: a qualitative study. J Clin Nurs. 2014;23(21–22):3188–96. doi: 10.1111/jocn.12565 25453123

[43] Glasgow RE, Vogt TM, Boles SM. Evaluating the public health impact of health promotion interventions: the RE-AIM framework. Am J Public Health. 1999;89(9):1322–7. doi: 10.2105/ajph.89.9.132 PubMed 10474547 PMC1508772

[44] Hemming K, Haines TP, Chilton PJ, Girling AJ, Lilford RJ. The stepped wedge cluster randomised trial: rationale, design, analysis, and reporting. BMJ. 2015;350:h391. doi: 10.1136/bmj.h391 25662947

[45] Mdege ND, Man MS, Taylor Nee Brown CA, Torgerson DJ. Systematic review of stepped wedge cluster randomized trials shows that design is particularly used to evaluate interventions during routine implementation. J Clin Epidemiol. 2011;64(9):936–48. doi: 10.1016/j.jclinepi.2010.12.003 21411284

[46] Vanstone M, Neville TH, Clarke FJ, Swinton M, Sadik M, Takaoka A, et al. Compassionate End-of-Life Care: Mixed-Methods Multisite Evaluation of the 3 Wishes Project. Ann Intern Med. 2019. doi: 10.7326/M19-2438 PubMed . 31711111

[47] Perez Jolles M, Lengnick-Hall R, Mittman BS. Core Functions and Forms of Complex Health Interventions: a Patient-Centered Medical Home Illustration. J Gen Intern Med. 2019;34(6):1032–8. doi: 10.1007/s11606-018-4818-7 Epub 2019/01/10 30623387 PMC6544719

[48] Damschroder LJ, Aron DC, Keith RE, Kirsh SR, Alexander JA, Lowery JC. Fostering implementation of health services research findings into practice: a consolidated framework for advancing implementation science. Implement Sci. 2009;4:50. doi: 10.1186/1748-5908-4-50 19664226 PMC2736161

[49] Kleinheksel AJ, Rockich-Winston N, Tawfik H, Wyatt TR. Demystifying Content Analysis. Am J Pharm Educ. 2020;84(1):7113. doi: 10.5688/ajpe7113 32292185 PMC7055418

[50] Hsieh H-F, Shannon SE. Three approaches to qualitative content analysis. Qual Health Res. 2005;15(9):1277–88. doi: 10.1177/1049732305276687 16204405

[51] Casarett D, Pickard A, Bailey FA, Ritchie CS, Furman CD, Rosenfeld K, et al. A nationwide VA palliative care quality measure: the family assessment of treatment at the end of life. J Palliat Med. 2008;11(1):68–75. doi: 10.1089/jpm.2007.0104 18370895

[52] Teno JM, Mor V, Ward N, Roy J, Clarridge B, Wennberg JE, et al. Bereaved family member perceptions of quality of end-of-life care in U.S. regions with high and low usage of intensive care unit care. J Am Geriatr Soc. 2005;53(11):1905–11. doi: 10.1111/j.1532-5415.2005.53563.x 16274371

[53] Hodde NM, Engelberg RA, Treece PD, Steinberg KP, Curtis JR. Factors associated with nurse assessment of the quality of dying and death in the intensive care unit. Crit Care Med. 2004;32(8):1648–53. doi: 10.1097/01.ccm.0000133018.60866.5f 15286539

[54] Walling AM, Asch SM, Lorenz KA, Wenger NS. Impact of consideration of transplantation on end-of-life care for patients during a terminal hospitalization. Transplantation. 2013;95(4):641–6. Epub 2012/12/01. doi: 10.1097/TP.0b013e318277f238 23197177

[55] US Department of Veterans Affairs Center for Health Equity Research and Promotion: The Bereaved Famiy Survey-Inpatient 2020 [cited 2022 Jan 3]. Available from: https://www.cherp.research.va.gov/PROMISE/The_PROMISE_Survey.asp.

[56] Zigmond AS, Snaith RP. The hospital anxiety and depression scale. Acta Psychiatr Scand. 1983;67(6):361–70. doi: 10.1111/j.1600-0447.1983.tb09716.x 6880820

[57] Blevins CA, Weathers FW, Davis MT, Witte TK, Domino JL. The Posttraumatic Stress Disorder Checklist for DSM-5 (PCL-5): Development and Initial Psychometric Evaluation. J Trauma Stress. 2015;28(6):489–98. doi: 10.1002/jts.22059 26606250

[58] Chang DW, Neville TH, Parrish J, Ewing L, Rico C, Jara L, et al. Evaluation of Time-Limited Trials Among Critically Ill Patients With Advanced Medical Illnesses and Reduction of Nonbeneficial ICU Treatments. JAMA Intern Med. 2021;181(6):786–94. doi: 10.1001/jamainternmed.2021.1000 33843946 PMC8042568

[59] Taich Z WN, Walling A, Bear D, Cook D, Tseng C, Neville T. The 3 Wishes Program Improves Families’ Experience of Emotional and Spiritual Support at the End-Of-Life. [Abstract]. In Press; 2022. 10.1007/s11606-022-07638-7 PMC9113739 35581456

[60] Roza KA, Lee EJ, Meier DE, Goldstein NE. A survey of bereaved family members to assess quality of care on a palliative care unit. J Palliat Med. 2015;18(4):358–65. doi: 10.1089/jpm.2014.0172 25793359

[61] Donnelly S, Prizeman G, Coimín DÓ, Korn B, Hynes G. Voices that matter: end-of-life care in two acute hospitals from the perspective of bereaved relatives. BMC Palliat Care. 2018;17(1):117. doi: 10.1186/s12904-018-0365-6 Epub 2018/10/21 30340568 PMC6195738

[62] Wall RJ, Engelberg RA, Gries CJ, Glavan B, Curtis JR. Spiritual care of families in the intensive care unit. Crit Care Med. 2007;35(4):1084–90. doi: 10.1097/01.CCM.0000259382.36414.06 17334245

[63] Maslach C, Jackson SE, Leiter MP. Maslach burnout inventory manual. 3rd ed. Palo Alto, Calif. (577 College Ave., Palo Alto 94306): Consulting Psychologists Press; 1996. p. iv, 52.

[64] Guest G, Johnson L. How Many Interviews Are Enough? An Experiment with Data Saturation and Variability. Field Methods. 18. Sage Publications; 2006. p. 59–82.

[65] Francis JJ, Johnston M, Robertson C, Glidewell L, Entwistle V, Eccles MP, et al. What is an adequate sample size? Operationalising data saturation for theory-based interview studies. Psychol Health. 2010;25(10):1229–45. doi: 10.1080/08870440903194015 20204937

[66] Hussey MA, Hughes JP. Design and analysis of stepped wedge cluster randomized trials. Contemp Clin Trials. 2007;28(2):182–91. doi: 10.1016/j.cct.2006.05.007 16829207

[67] Hughes JP, Granston TS, Heagerty PJ. Current issues in the design and analysis of stepped wedge trials. Contemp Clin Trials. 2015;45(Pt A):55–60. doi: 10.1016/j.cct.2015.07.006 26247569 PMC4639463

[68] Panagioti M, Panagopoulou E, Bower P, Lewith G, Kontopantelis E, Chew-Graham C, et al. Controlled Interventions to Reduce Burnout in Physicians: A Systematic Review and Meta-analysis. JAMA Intern Med. 2017;177(2):195–205. doi: 10.1001/jamainternmed.2016.7674 27918798

[69] Gaglio B, Henton M, Barbeau A, Evans E, Hickam D, Newhouse R, et al. Methodological standards for qualitative and mixed methods patient centered outcomes research. BMJ. 2020;371:m4435. doi: 10.1136/bmj.m4435 33361103 PMC7756351

[70] McLellan E, MacQueen KM, Neidig JL. Beyond the Qualitative Interview: Data Preparation and Transcription. Field Methods. 2003;15(1):63–84. doi: 10.1177/1525822x02239573

[71] MacQueen K ME, Kay K, Milstein B. Codebook development for team-based qualitative analysis. Cultural Anthropology Methods J. 1998;10:31–6.

[72] Saunders B, Sim J, Kingstone T, Baker S, Waterfield J, Bartlam B, et al. Saturation in qualitative research: exploring its conceptualization and operationalization. Qual Quant. 2018;52(4):1893–907. doi: 10.1007/s11135-017-0574-8 29937585 PMC5993836

[73] Morse WC, Lowery DR, Steury T. Exploring Saturation of Themes and Spatial Locations in Qualitative Public Participation Geographic Information Systems Research. Soc Natur Res. 2014;27(5):557–71. doi: 10.1080/08941920.2014.888791

[74] Anguera MT, Blanco-Villaseñor A, Losada JL, Sánchez-Algarra P, Onwuegbuzie AJ. Revisiting the difference between mixed methods and multimethods: Is it all in the name?. Qual Quant. 2018;52(6):2757–70. doi: 10.1007/s11135-018-0700-2

[75] Viera AJ, Garrett JM. Preliminary study of a school-based program to improve hypertension awareness in the community. Fam Med. 2008;40(4):264–70. Epub 2008/04/03; 18382839

[76] Kinder D, Smith D, Ersek M, Wachterman M, Thorpe J, Davis D, et al. Family reports of end-of-life care among veterans in home-based primary care: The role of hospice. J Am Geriatr Soc. 2022;70(1):243–50. doi: 10.1111/jgs.17486 34585735

