# Supplementary material for: Implementation and evaluation of the 3 Wishes Project in safety-net hospitals: Protocol for a hybrid effectiveness-implementation study
Source: PLoS One. 2025 May 2;20(5):e0320843. doi: 10.1371/journal.pone.0320843 (PMC12047788; doi:10.1371/journal.pone.0320843)
Supplement: S1 Protocol — (PDF) [file pone.0320843.s001.pdf]

Protocol: Personalized End of Life Care in Safety-Net hospitals: Implementation of the 3 Wishes Project

Principle Investigator: Thanh Neville, MD, MSHS

Sites and Site-PI's:

- Olive View Medical Center: Nadar Kamangar, MD
- Harbor UCLA: Hope Cassano, MD
- LAC+USC: Emily Beers, MD

Brief Background and Summary: Dignified and compassionate end-of-life (EOL) care is a cornerstone of high-quality, patient-centered care, but in safety-net hospitals EOL care is often overlooked, considered too late, or not at all. By eliciting and implementing final wishes for dying patients, the 3 Wishes Project (3WP) has demonstrated, in tertiary academic centers, that acts of compassion can improve the EOL experience and help families cope with loss. We propose to implement the 3WP in safety-net hospitals where there are less resources and more diverse, disadvantaged patient populations, and hypothesize that there will be similar positive effects on the EOL experience for patients, families, and clinicians.

Aim 1: Refine, tailor, and use the 3WP implementation strategy (via the online "3WP Toolkit") to overcome SNH-specific implementation barriers.

1. Site-PI's will email nursing, physician, and hospital volunteer listservs to recruit stakeholders to participate in short interviews after examining our online 3WP Toolkit website.
2. Interviews will be approximately 20 minutes along via Zoom to elicit feedback.
3. Feedback to 3WP will be incorporated into the 3WP Toolkit

Aim 2: Implement the 3WP in three SNHs and measure the 3WP effects on a) the quality of EOL ICU care and bereaved families' psychological symptoms and b) effect of 3WP on nurse burnout as compared to usual care. Start time at each site will be staggered by 2 months.

1. Obtain baseline data
    a. Six months prior to 3WP implementation, each site will start obtaining weekly reports of all inpatient deaths in the ICU in which 3WP is to be initiated
    b. Each ICU decedent during the study period will be characterized with data from the EHR. We will extract from the EHR the patient's age, sex, race, ethnicity, primary language, insurance status, co-morbidities, acute severity of illness score (SOFA or APACHE), length of stay, serious illness diagnosis, ICU diagnosis, utilization of ICU resources.
    c. Throughout the study period, research team will send after-death survey to families of ICU decedents 3 months after the patient's death.
    d. One month prior to 3WP, the site-PI will send an email to all ICU nurses with a link to the burnout survey to all nurses in the ICU.
2. 3WP Implementation starts after 6 months of data collection
    a. Site-PI's (with help of PI) will introduce 3WP to nurses and physicians and recruit champions
    b. Site-PI's (with help of PI) will arrange and lead unit-wide 3WP training sessions (likely one for day and one for night shift)
    c. Nurses and physicians will implement 3WP for dying patients and their families
    d. Document all 3WP patients, date of implementation, who the 3WP was implemented by, family name and contact, wishes implemented and their costs.
3. Evaluate the 3WP effects on bereaved families
    a. Continue after-death surveys
    b. Contact and interview 10-12 family members of patients whose care involved the 3WP at each hospital (interviews will be recorded, transcribed, and analyzed de-identified)
4. Evaluate the 3WP on nurses

   a. Nurses will be asked to complete the burnout surveys 6 months and one year after 3WP implementation
   b. One year after 3WP implementation, 10-12 nurses per hospital will be recruited and interviewed for feedback (interviews will be recorded, transcribed, and analyzed de-identified)

Aim 3: Evaluate 3WP implementation in SNHs.

1. Determine quantitative measurement of reach and adoption (determine the proportion of eligible patients who received 3WP, compare variations in rates of 3WP patient enrollment, proportion of clinician participation, and cost across the 3 hospitals)
2. Perform semi-structured interviews with about 15 clinicians/leaders (3-6 per hospital) to explain variations in implementation processes and outcomes (interviews will be recorded, transcribed, and analyzed de-identified)
